# Is There a Role for Hormonal Therapy in Men with Oligoasthenoteratozoospermia (OAT)?

**DOI:** 10.3390/jcm14010185

**Published:** 2024-12-31

**Authors:** Selahittin Çayan, Ahmet Barış Altay, Amarnath Rambhatla, Giovanni M. Colpi, Ashok Agarwal

**Affiliations:** 1 Department of Urology, Andrology Section, School of Medicine, University of Mersin, Mersin 33110, Turkey; 2 Global Andrology Forum, Moreland Hills, OH 44022, USA; ahmetbaris.altay@yahoo.com (A.B.A.); arambha1@hfhs.org (A.R.); gmcolpi@yahoo.com (G.M.C.); agarwaa32099@outlook.com (A.A.); 3 Department of Urology, Andrology Section, Faculty of Medicine, Ege University, İzmir 35040, Turkey; 4 Department of Urology, Henry Ford Health System, Vattikuti Urology Institute, Detroit, MI 48202, USA; 5 Andrology and IVF Center, Next Fertility Procrea, 6900 Lugano, Switzerland; 6 Cleveland Clinic, Cleveland, OH 44195, USA

**Keywords:** hormone, male infertility, oligoasthenoteratozoospermia, sperm, therapy

## Abstract

Hormonal factors play an essential role as an underlying causative factor of oligoasthenoteratozoospermia (OAT), and these patients can benefit from hormonal medications that modulate the hypothalamic–pituitary–gonadal axis. This review aims to outline the various medications used as hormonal therapy in treating infertile men with OAT. This manuscript focuses on essential hormonal evaluation, identifying men who would benefit from treatment, selecting the appropriate medication, determining the duration of therapy, and evaluating hormonal treatment outcomes. Additionally, novel markers that can broaden the horizon of hormonal treatment in infertile men with OAT are discussed. Hormonal-based therapy options in men with OAT include selective estrogen receptor modulators (SERMs), aromatase inhibitors (AIs), dopamine agonists, and injections such as gonadotropin-releasing hormone (GnRH) analogs and gonadotropins. Treatment duration and the expected success will dictate the final treatment type for couples. In conclusion, hormonal therapy may improve spermatogenesis in infertile men with low serum testosterone. Gonadotropins and SERMs may increase sperm parameters in men with infertility and normal serum gonadotropin levels. AIs might help improve spermatogenesis in infertile men with a total testosterone (ng/mL)/estradiol (pg/mL) ratio < 0.10. In addition, dopamine agonists may play a role in enhancing spermatogenesis in infertile men with hyperprolactinemia.

## 1. Introduction

Male factor infertility significantly contributes to approximately half of all cases of infertility in couples, with an estimated 40% of male infertility cases being idiopathic in origin [1]. Addressing these complex cases necessitates a multidisciplinary approach, which is fundamental in clinical practice for managing fertility issues. According to World Health Organization (WHO) 2010 criteria, oligoasthenoteratozoospermia (OAT) is defined as the presence of a sperm concentration of <15 million/mL, total sperm motility of <40%, progressive sperm motility of <32%, and normal sperm forms of <4% [2]. This review aims to provide an updated perspective on hormonal treatment strategies for men with OAT and to highlight recent advancements relevant to fertility specialists, urologists, and gynecologists.

Various conditions, including uncontrolled metabolic disorders such as obesity and diabetes mellitus, severe systemic illnesses, infections, and inflammatory diseases, can disrupt the hypothalamic–pituitary–gonadal (HPG) axis. This disruption often results in reduced testosterone levels, while gonadotropin levels may remain relatively normal, leading to poor sperm parameters [3].

The patient population with OAT represents a heterogeneous group with a wide range of etiopathogenic factors, including endocrine disruptors, infections, varicocele, tumors, trauma, systemic diseases, medications, lifestyle factors, obesity, smoking, alcohol use, and hormonal imbalances. In the pathogenesis of hormonally mediated infertility, both congenital factors (such as Kallmann syndrome) and acquired factors are implicated. Numerous medications, including anti-androgenic agents (cimetidine, spironolactone, ketoconazole, flutamide), corticosteroids, and anabolic agents, have been linked to impaired spermatogenesis. Obesity, as a body mass index of >30 kg/m^2^, is associated with low serum testosterone and sex hormone-binding globulin (SHBG) levels despite normal follicle-stimulating hormone (FSH) and luteinizing hormone (LH) levels, a condition known as functional hypogonadism. Additionally, endocrine-disrupting chemicals, such as heavy alcohol consumption, can adversely affect spermatogenesis by impairing Leydig cell function [4]. Testicular tumors that secrete beta-human chorionic gonadotropin (hCG) or hormone-secreting tumors (adrenocortical or Leydig cell tumors) can exert negative feedback on the hypothalamic-pituitary axis, further complicating the hormonal landscape [5].

Hormonal balance plays a pivotal role in the progress of OAT, and patients may benefit from targeted hormonal modulation. An average level of intratesticular testosterone is critical for spermatogenesis, particularly for the progression of round spermatids to mature sperm. A significant reduction in testosterone levels can lead to impaired spermatogenesis and the subsequent development of OAT [6]. Although testosterone replacement therapy (TRT) is commonly recommended for male hypogonadism, the administration of exogenous synthetic testosterone can negatively impact the HPG axis, leading to suppressed gonadotropin-releasing hormone (GnRH) and gonadotropin secretion. This suppression decreases intratesticular testosterone levels and impairs spermatogenesis [3,7,8,9]. Consequently, men who desire to preserve their fertility potential should avoid exogenous synthetic testosterone therapy. The effect of intratesticular testosterone on the primary or secondary spermatocyte is not crucial for the completion of male meiosis. However, its influence on Sertoli cells is vigorous for the completion of spermatogenesis.

Hormonal treatments aimed at increasing intratesticular testosterone through androgen receptor pathways in Sertoli cells, peritubular myoid cells, Leydig cells, and fibroblasts have shown promise in enhancing spermatogenesis. Notably, studies in androgen receptor knockout mice have demonstrated that spermatogenesis can be halted at the stages of meiosis and spermiogenesis, underscoring the critical role of testosterone in the initiation and maintenance of spermatogenesis [10]. However, the optimal intratesticular testosterone level required to stimulate spermatogenesis remains unclear.

This review provides a comprehensive overview of hormonal evaluation, focusing on identifying candidates for hormonal treatment, selecting appropriate therapies, determining treatment duration, and evaluating treatment outcomes. Additionally, we address ongoing controversies in hormone therapy for OAT patients and explore novel biomarkers that may expand the scope of hormonal treatment in infertile men with OAT.

### 1.1. HPG Axis and Its Relevance

The HPG axis is the maestro for regulating the spermatogenesis process. The hypothalamus secretes gonadotropin-releasing hormone (GnRH) in a pulsatile fashion, which circulates via the hypothalamic–hypophyseal portal to reach the anterior pituitary gland, where it stimulates FSH and LH secretion. It is well known that FSH acts on Sertoli cells to stimulate spermatogenesis, and LH acts on Leydig cells to increase testosterone production. The negative feedback mechanism works with the aromatization of testosterone to estradiol (E2) and decreases GnRH, LH, and FSH [11]. HPG and hypothalamic–pituitary–somatotropic (HPS) axes have excellent communication such that growth hormone (GH) strongly stimulates sex hormones during puberty, and after late adulthood, their secretion decreases. Also, IGF-1 (insulin-like growth factor) has a significant role during Tanner stages I to III, and after testicular volume increases, IGF-1 secretion decreases. GH secretion and receptor sensitivity are closely related to sex hormones; however, testosterone and E2 control GH secretion at the central and peripheral levels and inhibit IGF-1 production [12].

### 1.2. Drugs Used for Hormonal Therapy and Specific Situations in Men with OAT

1. Gonadotropins
2. GnRH analogues
3. Selective estrogen receptor modulators (SERMs)
4. Aromatase inhibitors (AIs)
5. Dopamine agonists

In infertile men with OAT, the rationale for hormone-based treatment is to stimulate endogenous testosterone production and optimize intratesticular testosterone levels, leading to improvement in spermatogenesis [13]. Various hormonal-based pharmacological agents have been used empirically to induce spermatogenesis in treating infertile men with OAT. Table 1 shows these pharmacological agents and their dosages to induce spermatogenesis. These hormonal therapies include GnRH, hCG, human menopausal gonadotropin (hMG), purified/highly purified FSH (pFSH), recombinant FSH (rFSH), SERMs, and AIs [3,7,8,9,14,15,16,17,18]. SERMs such as clomiphene citrate, tamoxifen, toremifene, or raloxifene and AIs such as letrozole or anastrozole are prescribed off-label. However, they are relatively safe alternatives for the treatment of functional hypogonadism to help boost spermatogenesis and testosterone levels.

### 1.3. Gonadotropin Agents

The hormonal basis of spermatogenesis is regulated primarily by FSH- and LH-driven testosterone. Gonadotropins such as hCG, hMG, pFSH, and rFSH have been used to stimulate spermatogenesis in men with OAT by directly acting on the testes, thereby enhancing the production of testosterone and sperm. Therefore, the primary therapeutic role of gonadotropins is to increase sperm production in synergy with intratesticular testosterone. These agents are reported to have a significantly higher spontaneous pregnancy rate (16%) compared to controls (7%), as reported in a Cochrane review [14]. A meta-analysis by Santi et al. analyzed 15 studies including 1275 men (half of the men treated with FSH and half of the men treated with placebo or untreated) and reported that FSH treatment provided significant increases in pregnancy rates spontaneously or with the use of assisted reproductive technology (ART) and an increase in sperm concentration in men with various severity of OAT [19]. In a recent systematic review and meta-analysis, Zhang et al. included 12 randomized controlled studies and one retrospective case-control study, consisting of 924 males with OAT, to compare conventional sperm parameters and also sperm DNA fragmentation index (SDF) between the men receiving FSH and the control group [20]. They found a significant increase in sperm concentration (MD: 4.52 million/mL, *p*-value of 0.004) and percentage of normal forms (MD: 2.87%, *p*-value of 0.04) and a significant decrease in SDF (MD: −12.62%, *p*-value of 0.002).

Both urinary and recombinant gonadotropin preparations are currently used in clinical practice. Prolonged treatment with urinary hCG and FSH may lead to the development of gonadotropin antibodies in infertile men. Thus, this situation may deteriorate gonadotropic signaling, leading to no significant improvement in spermatogenesis. However, recombinant formulations may rescue from immunologic binding and permit longer term spermatogenic signaling [21].

However, recombinant FSH is preferred due to its superior safety profile and higher quality. Despite these advantages, its use is often constrained by cost, inconvenience, and the absence of widely accepted standardized protocols [7,8].

FSH alone binds to FSH receptor (FSHR), stimulates Sertoli cells, and regulates mitotic proliferation. It can directly initiate spermatogenesis and has been shown to maintain spermatogenesis independent of testosterone. hCG, an analog of LH, stimulates Leydig cells via luteinizing hormone/choriogonadotropin receptors to produce testosterone. Like FSH, recombinant hCG is considered safer and better quality than its urinary counterpart. Exogenous gonadotropins, such as FSH and hCG, mimic the physiological actions of endogenous FSH and LH to promote spermatogenesis, with hCG treatment facilitating spermatogonial DNA synthesis. Gonadotropins are particularly indicated in the treatment of men with hypogonadotropic hypogonadism.

Gonadotropins like hCG, hMG, recombinant FSH, and recombinant LH have demonstrated significant improvements in sperm parameters and pregnancy rates when used to treat idiopathic male infertility, particularly in the context of ART [14,18]. Nevertheless, the efficacy of gonadotropin treatment remains controversial because of a limited number of placebo-controlled studies and, in theory, should be more effective for men with hypogonadism compared to idiopathic infertility. Furthermore, variability in gonadotropin preparations, treatment protocols, and duration across studies adds complexity.

Although the professional society guidelines do not recommend testosterone monotherapy for infertile men who try to conceive, some clinicians incorrectly believe that testosterone replacement therapy may improve sperm parameters in infertile men [1]. This belief is based on the limited number of high-quality studies reporting sperm parameters in infertile men with a history of testosterone use. In a recent study by Stocks et al., 77 infertile men who had prior exogenous testosterone use were treated with 3000 IU of hCG and 75 IU of FSH three times a week [21]. Of the patients, 74% demonstrated significant improvement in their sperm parameters. They also found no difference between men with no testosterone therapy and men with concurrent testosterone therapy during the hCG and FSH therapy. They concluded that concurrent testosterone treatment does not impede gonadotropin-mediated spermatogenic recovery.

In cases where fertility is the primary goal, gonadotropins serve as a viable alternative to TRT. However, the use of gonadotropin agents in infertile males with OAT is still in the stage of empirical treatment, and the professional society guidelines have not achieved a consensus on the therapeutic effect of gonadotropin agents in these OAT populations, especially in the presence of normal serum FSH levels.

In addition, they require frequent injections and necessitate strict monitoring by specialists to avoid potential downstream side effects. Its safety has been confirmed; however, acne, fatigue, flu-like symptoms, testicular pain, and gynecomastia are side effects of gonadotropin treatment [19,22].

Infertile men with larger testis volumes, higher pre-treatment sperm parameters, and lower serum FSH levels achieve a higher positive response to gonadotropin treatment. Published studies have not demonstrated the potential efficacy of this hormonal empirical treatment in men with idiopathic infertility and basal FSH levels higher than 8 mIU/mL [23]. Optimal dosing, timing, and treatment duration of gonadotropins in the treatment of infertile men with OAT have always been controversial. Therefore, clinicians should always monitor serum gonadal hormone levels and sperm parameters during a 3-month interval under the gonadotropin treatment. In a meta-analysis, Cannarella et al. investigated the efficacy of FSH dosing in men with idiopathic OAT and reported posttreatment outcomes according to FSH dosing regimens [24]. They found that a low-dosing FSH regimen demonstrated improvement only in sperm motility. However, a high-dosing regimen demonstrated significant improvement in sperm concentration, motility, and total sperm count. Therefore, serum gonadal hormone levels and conventional sperm parameters should be checked every 3 months up to 12 months, and the treatment may be stopped if pregnancy is not achieved.

### 1.4. GnRH Analogues

GnRH is secreted in a pulsatile manner and is regulated by serum testosterone levels. Infusion of GnRH stimulates LH and FSH expression. LH is stimulated with high-frequency pulses, and FSH expression is stimulated with low-frequency pulses. GnRH, a decapeptide, can be administered by intravenous (IV), intramuscular (IM), subcutaneous (SC), nasal, or sublingual routes; however, IV is the preferred method for the efficient, episodic release of LH and FSH. For the treatment of congenital hypogonadotropic hypogonadism, pump-administered pulsatile GnRH treatment is the preferred treatment choice [25]. Hot flushes, gynecomastia, erectile dysfunction, and decreased libido are possible side effects of GnRH analogs.

### 1.5. Selective Estrogen Receptor Modulators (SERMs)

Estrogen has been shown to regulate the reabsorption of luminal fluid in the head of the epididymis, and disruption of this function leads to the entry of sperm diluted in the epididymis rather than concentrated, resulting in infertility. Because estrogens exert negative feedback on gonadotropin secretion, various antiestrogens have been tried for infertility treatment, with the rationale that the absence of feedback inhibition will lead to increased secretion of gonadotropins. Clomiphene citrate is a SERM that increases the expression of gonadotropins. SERMs target central stimulation of FSH and LH, improving testosterone and spermatogenesis. In a systematic review and meta-analysis conducted with 16 controlled and non-controlled studies, Cannarella et al. demonstrated that 50 mg clomiphene citrate or 20–30 mg tamoxifen daily significantly increased sperm density, serum FSH, LH, testosterone levels, and pregnancy rates. However, there was no impact on progressive and total motility compared to the control group [15]. In a recent systematic review and meta-analysis by Huijben et al., clomiphene citrate with approximately five months of treatment provided significantly improved sperm density, with a mean difference of 8.38 million; sperm motility, with a mean difference of 8.14%; and significant increases in serum FSH, LH, and total testosterone level (mean difference of 2.05 ng/mL) [16]. In various studies, treatment with clomiphene citrate provided a mean pregnancy rate of 17%, ranging from 0 to 40%. Mild side effects with clomiphene citrate treatment included headache, gynecomastia, dizziness, mood changes, and fatigue ranging from 0 to 27% [16]. In addition, venous thromboembolism risk during long-term treatment with tamoxifen has been reported [15].

### 1.6. Aromatase Inhibitors (AIs)

The androgen-to-estrogen ratio plays a crucial role in normal sexual growth and reproductive function in men. Aromatase is an enzymatic complex localized in the endoplasmic reticulum of various tissues that irreversibly transforms androgens into estrogens. Its activity can be demonstrated in several tissues, including the ovaries, brain, placenta, adipose tissue, muscle, liver, breast, and estrogen-dependent breast cancer. Aromatase is involved in sexual differentiation, lipid metabolism, and bone structures, as well as in cancer development, which therefore suggests a major role for that enzyme [26]. Aromatase inhibitors (AIs) are divided into two distinct structural classes: steroidal, such as testolactone, and non-steroidal, such as anastrozole and letrozole [26]. These inhibitors increase endogenous testosterone levels and prevent the conversion of testosterone to estradiol via the aromatase enzyme. The total testosterone-to-estradiol ratio is calculated as total testosterone divided by estradiol. Candidates for AI therapy are typically men with a serum total testosterone (ng/mL)-to-estradiol (pg/mL) ratio of less than 0.10.

Non-steroidal AIs, such as anastrozole and letrozole, have been shown to effectively elevate peripheral serum testosterone levels, potentially enhancing sperm retrieval and fertilization rates in intracytoplasmic sperm injection (ICSI) procedures.

A meta-analysis consisting of 10 studies demonstrated that treatment with letrozole or anastrozole significantly increased sperm density, total sperm count, and serum FSH, LH, and total testosterone levels. However, the adverse effects associated with AI therapy, reported in 15.2% of cases, include decreased libido, osteopenia, liver dysfunction, skin rash, and headache during the prolonged treatment [17]. It is important to note that AI treatment often extends beyond six months, and the anticipated clinical improvements in semen parameters may not be substantial, particularly in men diagnosed with azoospermia [3,7].

In a separate meta-analysis encompassing eight studies with 517 patients, Del Giudice et al. reported that both steroidal (testolactone) and non-steroidal (anastrozole, letrozole) AIs significantly improved all evaluated hormonal and seminal outcomes, with a favorable safety profile [27]. However, further prospective randomized studies focused on such a selected category of infertile patients would be necessary before implementing aromatase inhibitors in clinical practice.

### 1.7. Dopamine Agonists

Prolactinoma, a pituitary adenoma, is most common in young cohorts. Medical management with dopamine antagonists is generally effective, with dopamine agonist resistance being around 10% with cabergoline and 20–30% with bromocriptine. The dopamine agonists of short-acting bromocriptine, with a dose of 2.5–10 mg daily, and long-acting cabergoline, with a dose of 0.5 mg twice weekly, are indicated to decrease serum prolactin levels and pituitary adenoma size in treating infertile men with hyperprolactinemia. Serum prolactin and testosterone levels should be monitored during and after the treatment. If dopamine agonists do not provide improvement in sperm parameters, other causes of male factor infertility should be investigated [28]. In dopamine agonist-resistant cases, AIs can be added to help enhance testosterone levels when hypogonadism persists [29]. A recent study evaluating 51 patients with prolactinoma treated with cabergoline showed improvement in sperm count, motility, and volume parameters after two years of treatment. In the short term, only semen volume and sperm density were increased [30]. Side effects include orthostatic hypotension, mood changes, nausea, and cardiac arrhythmia [20].

### 1.8. Recommendations According to Professional Society Guidelines

The European Association of Urology (EAU) 2024 guidelines suggest using gonadotropins, SERMS, and AIs in men with OAT with a weak strength rating of recommendations [1]. The same guidelines also suggested dopamine agonists in the presence of hyperprolactinemia with a weak strength rating of recommendations to improve spermatogenesis.

The American Urological Association (AUA)/the American Society for Reproductive Medicine (ASRM) guidelines suggest the use of AIs, hCG, SERMs, or a combination for infertile men with low serum testosterone with a grade C recommendation [31]. Men with idiopathic infertility should be informed about the limited benefits of SERMs for ART results (expert opinion). In addition, the clinician may consider treatment using gonadotropins for improving sperm concentration, pregnancy, and live-birth rates in men with idiopathic infertility with a grade B conditional recommendation.

## 2. Clinical Case Scenarios for the Clinicians

### 2.1. Case Presentation 1

An infertile couple who has been trying to conceive for 3 years presented to infertility clinics. The female partner (30 y.o.) had a regular gyno-endocrinologic evaluation. The male partner (33 y.o.) had no correctable pathology on physical examination, and his conventional semen analysis showed an ejaculate volume of 2.4 mL, a sperm concentration of 8 million/mL, and a progressive sperm motility (a + b) of 20%, with a total motile sperm count (TMSC) of 3.84 million. His serum hormone levels revealed an FSH level of 5.4 mIU/mL, an LH level of 4.8 mIU/mL, and a serum total testosterone level of 4.1 ng/mL. The question would be whether hormonal therapy should be recommended to the male to improve semen parameters.

#### Recommendations

This couple needs an intra-cytoplasmic sperm injection (ICSI) to achieve pregnancy with a sperm concentration of 8 million/mL and a progressive sperm motility (a + b) of 20% with a TMSC of 3.84 million. For couples who want to conceive naturally, empirical gonadotropin therapy (hCG or FSH) may improve sperm parameters, leading to increased spontaneous pregnancy, or down-stage or shift the level of assisted reproductive technologies (ARTs) (for example, from ICSI to intrauterine insemination, IUI).

### 2.2. Case Presentation 2

An infertile couple (39 y.o. male and 34 y.o. female) that has been trying to conceive for two years and had 1x intrauterine insemination (IUI) failure presented to infertility clinics. The male partner has an OAT with a TMSC of 5.4 million (ejaculate volume of 3 mL, sperm concentration of 10 million/mL, sperm motility (a + b) of 18%, and normal sperm forms of 3%). He had no palpable varicocele or male accessory gland infection, and his SDF level was 12% by TUNEL assay (reference range 0 to 30%). His serum hormone levels revealed an FSH level of 4.2 mIU/mL, an LH level of 2.8 mIU/mL, and a serum total testosterone level of 2.8 ng/mL. The female partner revealed no abnormality in the gyno-endocrinologic evaluation. The question is whether the male patient should be advised on SERMs such as clomiphene citrate to improve conventional sperm parameters and also to increase serum total testosterone level or whether an IVF cycle should be offered to the couple directly.

#### Recommendations

The couple should be counseled for both, emphasizing the pros and cons of each treatment plan. SERMs, particularly clomiphene, are recognized as effective and safe empirical therapies for idiopathic male infertility, especially in hypogonadal men. Clomiphene citrate therapy for at least three months might improve sperm parameters and increase testosterone levels, possibly leading to down-staging or shifting the level of ART and improving the chances of success for pregnancy. It is always necessary to advise improvement in lifestyle changes, as it is frequently a reason for functional hypogonadism in the presence of OAT.

### 2.3. Case Presentation 3

An infertile couple who has been trying to conceive for 2 years presented to infertility clinics. The female partner (32 y.o.) had a regular gyno-endocrinologic evaluation. The male partner is 35 years old and has severe obesity with no correctable pathology on physical examination. His body mass index (BMI) was 31 (normal range < 25). His conventional semen analysis showed an ejaculate volume of 2 mL, a sperm concentration of 12 million/mL, and a progressive sperm motility (a + b) of 20%, with a TMSC of 4.8 million. His serum hormone levels revealed an FSH level of 4.6 mIU/mL, an LH level of 3.8 mIU/mL, a serum total testosterone level of 2.1 ng/mL, and an estradiol level of 45 pg/mL. His total testosterone-to-estradiol ratio was calculated as 0.04, which was less than 0.10. The question is whether hormonal therapy should be recommended to the male to improve semen parameters and also to increase serum total testosterone level, which is less than the normal range.

#### Recommendations

This couple needs one of the ARTs (ICSI) to achieve pregnancy with a sperm concentration of 12 million/mL and progressive sperm motility (a + b) of 20% with a TMSC of 4.8 million. Men with a higher BMI have also exhibit deteriorated quantity and quality of sperm. For couples who want to conceive naturally, one of the aromatase inhibitors may improve sperm parameters and testosterone levels, leading to the possibility of spontaneous pregnancy or down-staging or shifting the level of ART (for example, from ICSI to IUI). It is always necessary to advise improvement in lifestyle changes, as it is frequently a reason for functional hypogonadism in the presence of OAT.

## 3. Future Considerations

The lack of standardization of hormonal treatment for patients with OAT is a clinical problem for urologists. Neither the EAU nor the AUA/ASRM guidelines strongly recommend hormonal therapy in patients with OAT. Examining the synergy of ITT and FSH might have important clues in daily practice because men with idiopathic OAT have no remarkable endocrinological laboratory values. Some markers, such as insulin-like factor 3, anti-Müllerian hormone, inhibin B, and 17-alpha-hydroxyprogesterone, have been investigated to predict intratesticular testosterone levels [32]. Also, FSH receptor polymorphism might affect the response of gonadotropins [33]. Instead of empirical hormonal treatment for male infertility, a specific agent for stimulating the spermatogenetic process will be innovative for clinicians. A new era will begin with personalized medicine leading the way [23].

Another issue explaining why some infertile men with OAT do not have positive responses to hormonal therapy is that about 30% of oligozoospermic men have partial epididymal obstruction, showing normal testicular volume and serum FSH levels as well as normal spermatogenesis at testis biopsy [34]. This pathology can be differentiated with a scrotal ultrasound, and a timely and accurate diagnosis may provide the appropriate treatment and avoid unnecessary hormonal therapy to enhance sperm production in men with OAT in the presence of partial epididymal obstruction. However, further studies are needed to understand the importance of partial blockage in the epididymis in infertile oligozoospermic men.

## 4. Conclusions

Treatment of male infertility involves a thorough assessment and appropriate, targeted therapies. Hormonal therapy may enhance spermatogenesis in men with OAT and low serum testosterone. Gonadotropins and SERMs can potentially increase sperm parameters in men with idiopathic infertility with a standard range of serum gonadotropin levels. However, better evidence is needed before they are routinely recommended in this setting. AIs may recover spermatogenesis in infertile men with a total testosterone (ng/mL)/estradiol (pg/mL) ratio < 0.10. In addition, dopamine agonists can be used to enhance spermatogenesis in infertile men with hyperprolactinemia.

## Figures and Tables

**Table 1:** Various hormonal-based pharmacological agents with different dosages induce spermatogenesis.

Treatment Agents	Suggested Dosages
**Gonadotropins**	
Human chorionic gonadotropin (hCG)	1500–5000 IU, 2–3 times/week, subcutaneously
Recombinant hCG	250 IU, two times/week, subcutaneously
Urinary FSH	75–150 IU, 2–3 times/week, subcutaneously
Recombinant FSH	75–150 IU, 2–3 times/week, subcutaneously
Human menopausal gonadotropin (hMG)	75–150 IU, 2–3 times/week, subcutaneously
**Antiestrogens**	
Clomiphene citrate	25–50 mg, two times/day, orally
Tamoxifen	10 mg, two times/day, orally
**Aromatase inhibitors**	
Anastrozole	1 mg, one time/day, perorally
Letrozole	2.5 mg, one time/day, perorally

## Abbreviations

Ais	Aromatase inhibitors
ART	Assisted reproductive technology
FSH	Follicle-stimulating hormone
GH	Growth hormone
GnRH	Gonadotropin-releasing hormone
hCG	Human chorionic gonadotropin
hMG	Human menopausal gonadotropin
HPG	Hypothalamic–pituitary gonadal
HPS	Hypothalamic–pituitary–somatotropic
IGF	Insulin-like growth factor
LH	Luteinizing hormone
OAT	Oligoasthenoteratozoospermia
pFSH	Purified/highly purified FSH
rFSH	Recombinant follicle-stimulating hormone
SERMs	Selective estrogen receptor modulators
TRT	Testosterone replacement therapy
